# Coeliac disease and type 2 diabetes risk: a nationwide matched cohort and Mendelian randomisation study

**DOI:** 10.1007/s00125-024-06175-8

**Published:** 2024-05-21

**Authors:** Shuai Yuan, Dan Leffler, Benjamin Lebwohl, Peter H. R. Green, Jiangwei Sun, Sofia Carlsson, Susanna C. Larsson, Jonas F. Ludvigsson

**Affiliations:** 1 Institute of Environmental Medicine, Karolinska Institutet, Stockholm, Sweden; 2 The Celiac Center at Beth Israel Deaconess Medical Center, Harvard Medical School, Boston, MA USA; 3 Department of Medicine, Celiac Disease Center at Columbia University Medical Center, New York, NY USA; 4 Departments of Medicine and Surgical Pathology, Columbia University College of Physicians and Surgeons, New York, NY USA; 5 Department of Medical Epidemiology and Biostatistics, Karolinska Institutet, Stockholm, Sweden; 6 Department of Surgical Sciences, Uppsala University, Uppsala, Sweden; 7 Department of Pediatrics, Orebro University Hospital, Orebro, Sweden

**Keywords:** Coeliac disease, Cohort, Mendelian randomisation, Type 2 diabetes

## Abstract

**Aims/hypothesis:**

While the association between coeliac disease and type 1 diabetes is well documented, the association of coeliac disease with type 2 diabetes risk remains undetermined. We conducted a nationwide cohort and Mendelian randomisation analysis to investigate this link.

**Methods:**

This nationwide matched cohort used data from the Swedish ESPRESSO cohort including 46,150 individuals with coeliac disease and 219,763 matched individuals in the comparator group selected from the general population, followed up from 1969 to 2021. Data from 9053 individuals with coeliac disease who underwent a second biopsy were used to examine the association between persistent villous atrophy and type 2 diabetes. Multivariable Cox regression was employed to estimate the associations. In Mendelian randomisation analysis, 37 independent genetic variants associated with clinically diagnosed coeliac disease at *p*<5×10^−8^ were used to proxy genetic liability to coeliac disease. Summary-level data for type 2 diabetes were obtained from the DIAGRAM consortium (80,154 cases) and the FinnGen study (42,593 cases).

**Results:**

Over a median 15.7 years’ follow-up, there were 6132 (13.3%) and 30,138 (13.7%) incident cases of type 2 diabetes in people with coeliac disease and comparator individuals, respectively. Those with coeliac disease were not at increased risk of incident type 2 diabetes with an HR of 1.00 (95% CI 0.97, 1.03) compared with comparator individuals. Persistent villous atrophy was not associated with an increased risk of type 2 diabetes compared with mucosal healing among participants with coeliac disease (HR 1.02, 95% CI 0.90, 1.16). Genetic liability to coeliac disease was not associated with type 2 diabetes in DIAGRAM (OR 1.01, 95% CI 0.99, 1.03) or in FinnGen (OR 1.01, 95% CI 0.99–1.04).

**Conclusions/interpretation:**

Coeliac disease was not associated with type 2 diabetes risk.

**Graphical Abstract:**

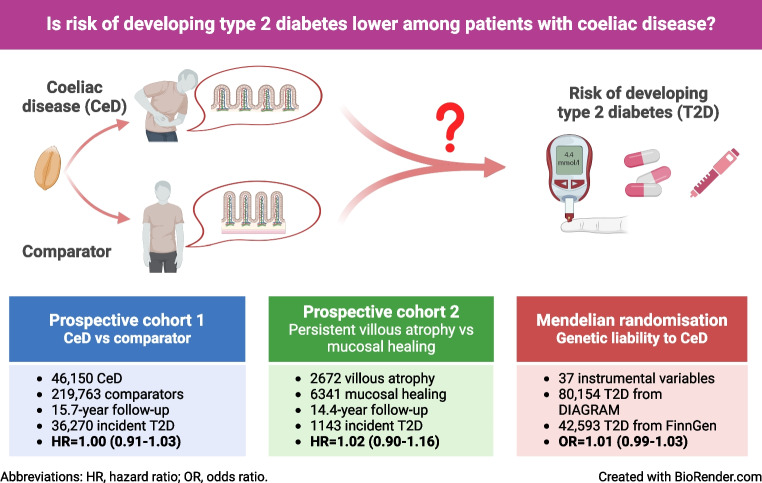

**Supplementary Information:**

The online version of this article (10.1007/s00125-024-06175-8) contains peer-reviewed but unedited supplementary material.


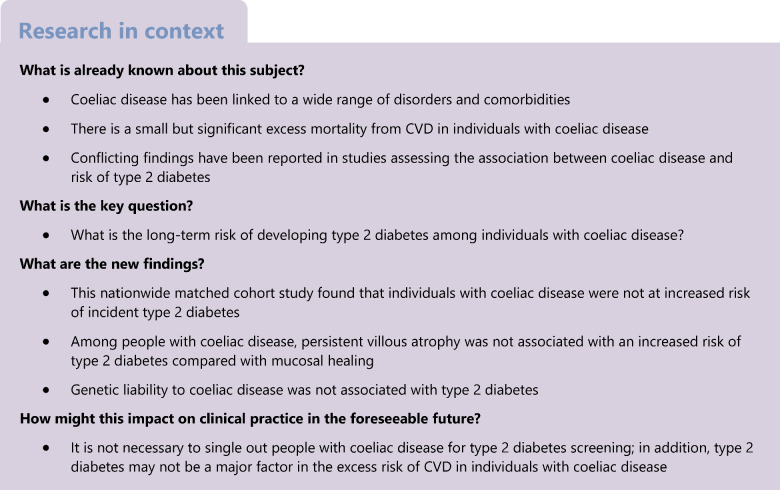


## Introduction

Coeliac disease is an immune-mediated disease characterised by small intestinal inflammation and villous atrophy [1]. Coeliac disease has been linked to a wide range of disorders and comorbidities including type 1 diabetes [2], thyroid disease [2], skin disease [3], certain cancers [4], but also overall death [5]. In the last decades, there has been an increasing interest in a possible association between coeliac disease and cardiometabolic disorders [6–9]. For example, our ESPRESSO (Epidemiology Strengthened by histoPathology Reports in Sweden) study suggests a small but significant excess mortality from CVD in people with coeliac disease [5]. However, the association between coeliac disease and risk of CVD has not been identified in Mendelian randomisation (MR) analysis [10, 11]. Likewise, conflicting data have been found for the association between coeliac disease and type 2 diabetes. Earlier data mostly show no [12] or an inverse [13] association. However, the two cited studies on type 2 diabetes were limited in size and did not study incident type 2 diabetes [12, 13]. The lack of long-term follow-up may be especially problematic since coeliac disease tends to have an early onset while type 2 diabetes occurs in middle and old age. Adding to this, one study [12] used different data sources for cases and controls making comparisons more difficult. A Swedish study identified a positive association between coeliac disease and type 2 diabetes risk in men [14]; however, the analysis was based on a rather small number of people with incident type 2 diabetes and had a comparatively short follow-up.

Given the limited and conflicting earlier data on coeliac disease and type 2 diabetes, we performed a longitudinal nationwide population-based study of 46,000 individuals with coeliac disease and 219,000 matched comparator individuals. To increase the robustness of our study, we performed a two-sample MR analysis to test whether genetic propensity for coeliac disease is associated with a risk of type 2 diabetes.

## Methods

### Study design

We first conducted a nationwide matched cohort study to examine the risk of incident type 2 diabetes among individuals with coeliac disease vs comparator individuals in the ESPRESSO cohort. To examine the impact of mucosal healing, we investigated the link between persistent villous atrophy and type 2 diabetes in participants with coeliac disease with at least two biopsies in the ESPRESSO cohort. Considering that most cases of type 2 diabetes were diagnosed prior to coeliac disease in the study by Kabbani et al [13], we also assessed the risk of type 2 diabetes before coeliac disease diagnosis or matching using a matched case–control design in ESPRESSO. For evidence triangulation, we used two-sample MR analysis to explore the association between genetic liability to coeliac disease, type 2 diabetes and four glycaemic traits. The cohort study received approval from the Stockholm ethics review board. In Sweden, informed consent is not needed for this cohort solely based on registries [15]. The MR analysis was based on summary-level data and thus requires no ethical approval or informed consent.

### Nationwide cohort study

The ESPRESSO cohort collects comprehensive gastrointestinal histopathology specimen records including information on personal identity number, date of biopsy, topography and morphology from all 28 pathology departments in Sweden [16]. We examined specimens submitted between 1969 and 2017, featuring topography codes specific to the small intestine (excluding the ileum) and Systematized Nomenclature of Medicine (SNOMED) codes corresponding to villous atrophy (see electronic supplementary material [ESM] Table 1). This strategy for the diagnosis of coeliac disease had been demonstrated to be accurate with a positive predictive value of 95% in a validation study using medical record review for a previous query of these data [17], with a positive predictive value of 99% in recent biopsy reports [5]. Subsequently, each person diagnosed with coeliac disease was matched with five comparator individuals from the general population by Statistics Sweden by birth year, sex, calendar year and county of residence. Moreover, comprehensive medical and demographic statistics pertaining to participants with coeliac disease and matched comparator individuals were obtained from diverse Swedish national registers, including the National Patient Register (NPR, with a positive predictive value for most disorders of 85–95% [18]), the Total Population Register, LISA (the Longitudinal integrated database for health insurance and labour market studies) and the Swedish Prescribed Drug Register (PDR). The unique personal identity number was used to integrate these datasets.

The outcome of the study was incident type 2 diabetes, which was defined by the ICD-7, 8, 9 and 10 codes from the NPR and the Anatomical Therapeutic Chemical (ATC) code from the PDR (ESM Table 2). Participants with any type of diabetes at baseline, pancreatitis, pancreatic insufficiency or pancreatic cancer were removed from the analysis. Participants were followed up from the index date (i.e. the diagnosis date for coeliac disease and matching date for comparator individuals) until the diagnosis of type 2 diabetes, death, emigration, or 31 December 2021. We censored follow-up for incident type 1 diabetes, other types of non-type 2 diabetes, insulin treatment within one month after first diabetes diagnosis, pancreatitis, pancreatic insufficiency or pancreatic cancer.

Given that mucosal healing status may influence the health of individuals with coeliac disease, we explored the association between persistent villous atrophy and type 2 diabetes. This analysis was conducted among 9888 people with coeliac disease who underwent a follow-up biopsy between 6 months and 5 years after their initial diagnosis. After removing individuals with no data from other registers, there were 2685 participants with coeliac disease with persistent villous atrophy and 6368 with mucosal healing. Those with coeliac disease were followed up from the date of the second biopsy until the diagnosis of type 2 diabetes, death, emigration, or 31 December 2021. The same approach was used for censoring.

Educational attainment, country of birth and the Charlson Comorbidity Index (CCI) [19] were included as covariates in all analyses based on ESPRESSO data. The baseline age was estimated by subtracting the birth year from the index year. Educational attainment, a proxy for socioeconomic status, was categorised into compulsory school (0–9 years), upper secondary (10–12 years), college or university (≥13 years) and unknown (missing data) [20]. The highest documented education level was referenced for each participant, while for children (<18 years old), we considered the highest educational attainment of their parents. Education data are available in >98% of all individuals aged 25–64 years with a high accuracy [20]. Data on country of birth (Sweden, other Nordic countries, or other countries [available from 1947 onward]) were obtained from the Total Population Register. The CCI, assessed up until 6 months prior to the index date, served as an indicator of overall health status [19].

### Nationwide matched case–control study

**Fig. 1:**
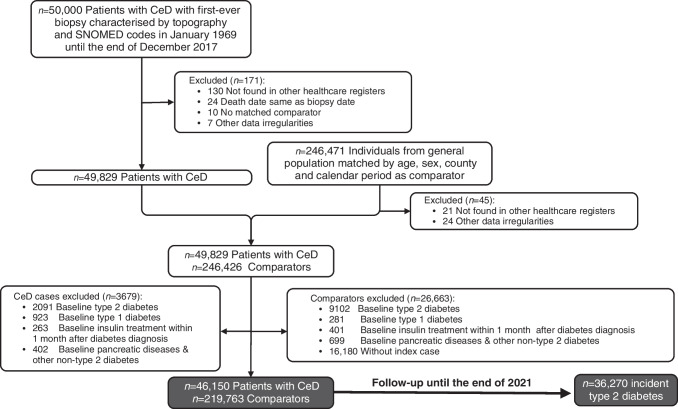
Flow chart of the selection of participants for the nationwide matched cohort. The participants were followed up to the end of 2021. CeD, coeliac disease. Image created with BioRender.com

Given that the majority of type 2 diabetes cases were diagnosed before coeliac disease in the study by Kabbani et al [13], we additionally conducted a matched case–control study to examine the risk of type 2 diabetes prior to coeliac disease diagnosis/matching based on ESPRESSO data. This analysis involved 49,829 individuals with coeliac disease and 246,426 control participants, before we further excluded those with baseline type 2 diabetes (Fig. 1). We removed participants with type 1 diabetes, other types of non-type 2 diabetes, insulin use within one month after first diabetes diagnosis, and pancreatic diseases prior to coeliac disease diagnosis to reduce bias. Type 2 diabetes status was also defined by ICD and ATC codes with data from NPR and PDR, albeit with the diagnostic date before coeliac disease diagnosis for individuals with coeliac disease and matching date for control participants. The same covariates were included in this case–control analysis.

### Two-sample MR study

Using genetic variants as instrumental variables for the exposure, MR analysis is a genetic data-based epidemiological approach that can strengthen causal inference by diminishing confounding and reverse causality. There are three important assumptions for MR analysis: (1) the genetic instruments should be robustly associated with coeliac disease; (2) the genetic instruments should not be associated with confounders; and (3) the genetic instruments should not be directly associated with type 2 diabetes or influence the risk of type 2 diabetes via alternative pathways.

We selected genetic instrumental variables (i.e. SNPs) to proxy the genetic liability to coeliac disease from a genome-wide meta-analysis of 12,041 individuals with coeliac disease and 12,228 control participants of European ancestry where coeliac disease cases were identified based on established clinical criteria, corroborative serological findings, and, universally, through small intestinal biopsy [21]. To satisfy the first assumption, SNPs associated with coeliac disease at the genome-wide significance level (*p*<5×10^−8^) were identified. To minimise the influence of linkage disequilibrium (i.e. genetic correlations between SNPs), we estimated the genetic correlation matrix of identified SNPs and removed SNPs in high linkage disequilibrium by setting *R*^*2*^ >0.001. Due to the complexity of linkage disequilibrium and to satisfy the third assumption, we removed SNPs located in the HLA gene region since HLA may directly impact type 2 diabetes risk [22, 23] and cause horizontal pleiotropy [24], leaving 37 SNPs used as the genetic instruments. We conducted a search in the PhenoScanner V2 database [25] for phenotypes linked to 37 SNPs to investigate the potential pleiotropy. This analysis revealed only a few autoimmune-related traits associated with 5–9 SNPs, suggesting a restricted range of pleiotropic effects. The *F* statistic (β^2^/standard error^2^) was estimated to measure the strength of the instrument [26] and found to be 68.8, indicating limited risk of weak instrument bias. Detailed information on SNPs used is shown in ESM Table 3.

Summary-level data for type 2 diabetes were obtained from the DIAGRAM (DIAbetes Genetics Replication And Meta-analysis) consortium (discovery) [27] and the FinnGen study R10 (replication) [28]. The DIAGRAM consortium conducted a multi-ancestry genome-wide meta-analysis among 180,834 individuals with type 2 diabetes and 1,159,055 control participants [27]. To minimise population structure bias, this MR analysis was based on data from the European populations only (80,154 case and 853,816 control participants). Associations were adjusted for age, sex, principal components and study-specific covariates [27]. FinnGen is an ongoing study combining genotype data from Finnish biobanks and digital health record data from Finnish health registries [28]. In the R10 data release, the study included 42,593 individuals with type 2 diabetes and 337,038 control participants with adjustment for age, sex, top ten principal components, FinnGen 1 or 2 chip or legacy genotyping batch as covariates. Detailed diagnosis of type 2 diabetes in the DIAGRAM consortium and FinnGen is displayed in ESM Table 4. We performed a secondary analysis to examine the association between genetic liability to coeliac disease and four glycaemic traits. In this analysis, summary-level data on fasting glucose, fasting insulin, 2 h glucose after an oral glucose challenge and HbA_1c_ were obtained from the Meta-Analyses of Glucose and Insulin-related traits Consortium (MAGIC) comprising up to 196,992 individuals without diabetes and of European ancestry [29]. No sample overlaps were found between the coeliac disease genome-wide association study (GWAS), the DIAGRAM consortium, the FinnGen study and MAGIC.

### Statistical analysis

In the cohort analysis, we first estimated the cumulative hazard of type 2 diabetes diagnosis between individuals with coeliac disease and comparator individuals. We then used Cox proportional hazards regression conditioned on matching factors (age, sex, county and calendar year) to estimate the association between coeliac disease and the risk of incident type 2 diabetes after adjustment for education levels, country of birth and the CCI. The proportional assumption was checked by Schoenfeld residual test and found to be satisfied (*p*=0.69). We performed sensitivity analyses by additionally excluding individuals with baseline diabetes insipidus and nephrogenic diabetes, gestational diabetes or Cushing’s syndrome. Following this, we conducted stratified analyses based on age, sex, start year of follow-up and follow-up duration. In this and all other analyses, data on sex were retrieved from the Total Population Register. Cox regression was also used to estimate the association between persistent villous atrophy and risk of type 2 diabetes among a subgroup of participants with a second biopsy. We performed a sensitivity analysis with additional adjustment for the time interval between two biopsies. That analysis was adjusted for age, sex, calendar year and county of residence, education levels, country of birth and the CCI.

The OR and corresponding 95% CI of type 2 diabetes among individuals with coeliac disease compared with control participants was estimated using conditional logistic regression. The analysis was adjusted for age, sex, calendar year and county of residence, education levels, country of birth and the CCI.

In MR analysis, we harmonised the data based on both effect and non-effect alleles. The inverse variance weighted method under the multiplicative random effects was used as the primary analysis and supplemented by the weighted median [30], MR-Egger [31] and MR-PRESSO [32] methods. The weighted median can provide a consistent estimate when >50% of SNPs are valid [30]. The MR-Egger can detect directional pleiotropy by its intercept test and generate estimate after adjusting for pleiotropy although the analysis is usually underpowered [31]. The MR-PRESSO analysis can detect SNP outliers and provide estimates after removal of identified outliers [32]. We calculated the Cochran’s *Q* to assess the heterogeneity between SNP estimates. For coeliac disease–type 2 diabetes association, we additionally used scatter plots to visually detect potential SNP outliers and estimated the associations after removal of the identified outliers.

Cox and conditional logistic regression analyses were conducted using R 4.4.1. Two-sample MR analysis was performed using the TwoSampleMR package in R 4.3.2 (https://github.com/MRCIEU/TwoSampleMR). Statistical significance was determined at a two-sided *p* value of ≤0.05.

## Results

### Nationwide cohort study

After exclusion of those without data from other registers, suitable comparator individuals, data irregularities, baseline type 2 diabetes, type 1 diabetes or other types of non-type 2 diabetes, insulin use within one month after diabetes diagnosis, and pancreatic diseases, we included 46,150 individuals with coeliac disease and 219,763 comparator individuals in the cohort analysis (Fig. 1). The mean age at diagnosis was 30.2 years, and 63.4% of participants were female. For participants with coeliac disease, 39.8% were less than 18 years old at the time of diagnosis, and 61.8% received their diagnosis after 1 January 2000. Compared with non-coeliac disease comparator individuals, participants with coeliac disease had a higher CCI (Table 1).

**Table 1:** Baseline characteristics of individuals with coeliac disease and comparator individuals

Characteristic	Coeliac disease *N*=46,150	Matched^a^ comparator group *N*=219,763
Female	29,108 (63.1)	139,432 (63.4)
Male	17,042 (36.9)	80,331 (36.6)
Age at diagnosis, years		
Mean	31.0±24.9	30.0±24.4
Median (IQR)	27 (7–52)	26 (7–50)
Range	0–95	0–95
Age groups		
<18 years	18,379 (39.8)	90,003 (41.0)
≥18–<40 years	10,376 (22.5)	50,180 (22.8)
≥40–<60 years	9364 (20.3)	44,898 (20.4)
≥60 years	8031 (17.4)	34,682 (15.8)
Country of birth^b^		
Sweden	43,220 (93.7)	196,867 (89.6)
Other Nordic country	1038 (2.2)	5086 (2.3)
Other country	1892 (4.1)	17,804 (8.1)
Level of education^c^		
≤9 years	9583 (23.8)	46,279 (24.3)
10–≤12 years	17,223 (42.9)	81,859 (43.0)
≥13 years	13,379 (33.3)	62,448 (32.8)
Parents’ highest education levels (used when patient's level of education is missing)^d^		
≤9 years	6767 (18.9)	32,403 (19.2)
10–≤12 years	14,901 (41.7)	70,588 (41.8)
≥13 years	14,089 (39.4)	65,720 (39.0)
Start year of follow-up		
1969–1989	4265 (9.2)	21,098 (9.6)
1990–1999	13,349 (28.9)	65,648 (29.9)
2000–2009	18,241 (39.5)	86,369 (39.3)
2010–2017	10,295 (22.3)	46,648 (21.2)
CCI		
0	37,451 (81.2)	195,858 (89.1)
1	4515 (9.8)	12,292 (5.6)
2	1716 (3.7)	5816 (2.6)
≥3	2468 (5.3)	5797 (2.6)

Data are expressed as *n* (%), mean±SD or median (IQR)

^a^Coeliac disease cases and comparator individuals were matched by birth year, sex, calendar year and county of residence

^b^Data missing for country of birth in six matched comparator individuals

^c^Data missing for level of education in 5965 individuals with coeliac disease and 29,177 matched comparator individuals

^d^Data missing for level of education in the parents of 10,393 individuals with coeliac disease and 51,052 matched comparator individuals

**Fig. 2:**
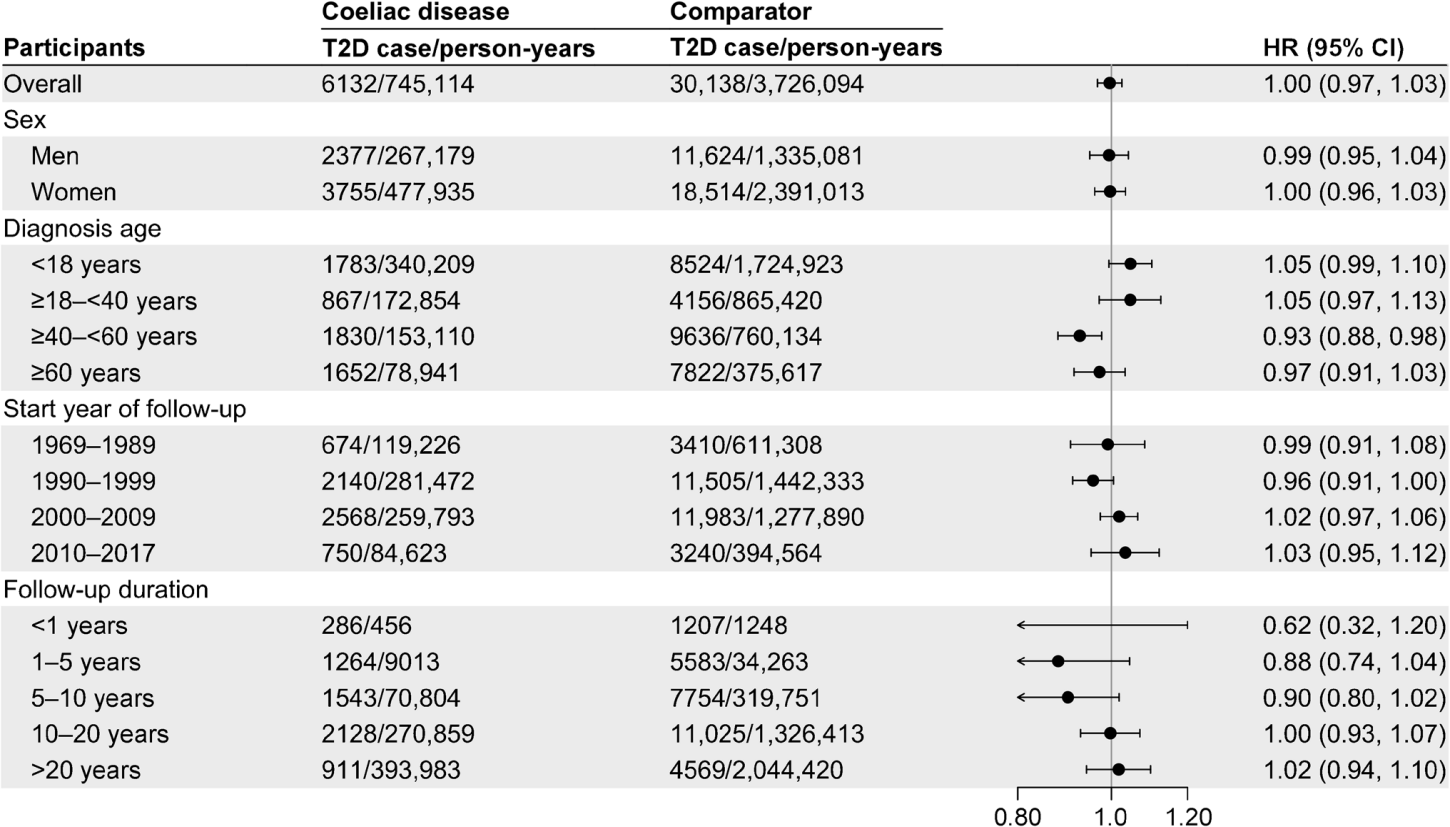
HR (95% CI) of incident type 2 diabetes (T2D) for individuals with coeliac disease compared with comparator individuals after a median 15.7-year follow-up. The associations were adjusted for age, sex, calendar year and county of residence, education levels, country of birth and CCI

During a median follow-up of 15.7 (IQR 9.9–23.0) years, there were 6132 (13.3%) incident type 2 diabetes cases among the participants with coeliac disease and 30,138 (13.7%) incident type 2 diabetes cases in the comparator group. The crude incidence rate of type 2 diabetes stood at 8.2 per 1000 person-years for participants with coeliac disease and 8.1 per 1000 person-years for comparator individuals, findings close to those in another Swedish study [33]. The Kaplan–Meier curve showed no significant difference in cumulative incidence of type 2 diabetes between individuals with coeliac disease and those in the comparator group across the follow-up period (ESM Fig. 1). For participants aged ≥40–<60 and ≥60 years, the cumulative incidence rate in both coeliac disease and the comparator group was around 1.0% and 1.9%, respectively, after a 15 year follow-up. After multivariable adjustment, individuals with coeliac disease were not at increased risk of type 2 diabetes with an HR of 1.00 (95% CI 0.97, 1.03; *p*=0.784) compared with their comparator individuals (Fig. 2). The null association persisted in the sensitivity analysis after additionally removing 653 individuals with baseline diabetes insipidus and nephrogenic diabetes, gestational diabetes, or Cushing’s syndrome (HR 1.00 [95% CI 0.97, 1.03]; *p*=0.841). Likewise, the null association between coeliac disease and the risk of type 2 diabetes was observed in analyses stratified by sex, start year of follow-up and follow-up duration (Fig. 2). However, there was an inverse association among individuals with coeliac disease diagnosis between 40 and 60 years (HR 0.93 [95% CI 0.88, 0.98]) but no association in other age groups when stratifying on age at coeliac disease diagnosis.

Among 9053 participants with a second biopsy followed for a median 14.4 (IQR 8.8–20.7) years, there were 398 (14.8%) incident type 2 diabetes cases among people with persistent villous atrophy and 745 (11.7%) in mucosal healing. The baseline features of individuals with persistent villous atrophy and mucosal healing are presented in ESM Table 5. The crude incidence rate was 9.6 and 7.7 per 1000 person-years for persistent villous atrophy and mucosal healing, respectively. After adjustment for covariates, persistent villous atrophy was not associated with an increased risk of type 2 diabetes (HR 1.02 [95% CI 0.90, 1.16]; *p*=0.756). This null association remained in a sensitivity analysis with additional adjustment for the time interval between two biopsies (HR 1.02 [95% CI 0.90, 1.16]) and stratified analyses (ESM Table 6).

### Nationwide matched case–control study

We included 45,903 coeliac disease cases and 220,849 matched controls in the case–control study. A total of 1901 (4.14%) coeliac disease cases and 7923 (3.59%) controls had a prior type 2 diabetes diagnosis, respectively. Compared with controls, coeliac disease cases had an OR of 1.04 (95% CI 0.98, 1.10; *p*=0.124) for prior type 2 diabetes in the multivariable conditional logistic regression.

### Two-sample MR study

**Fig. 3:**
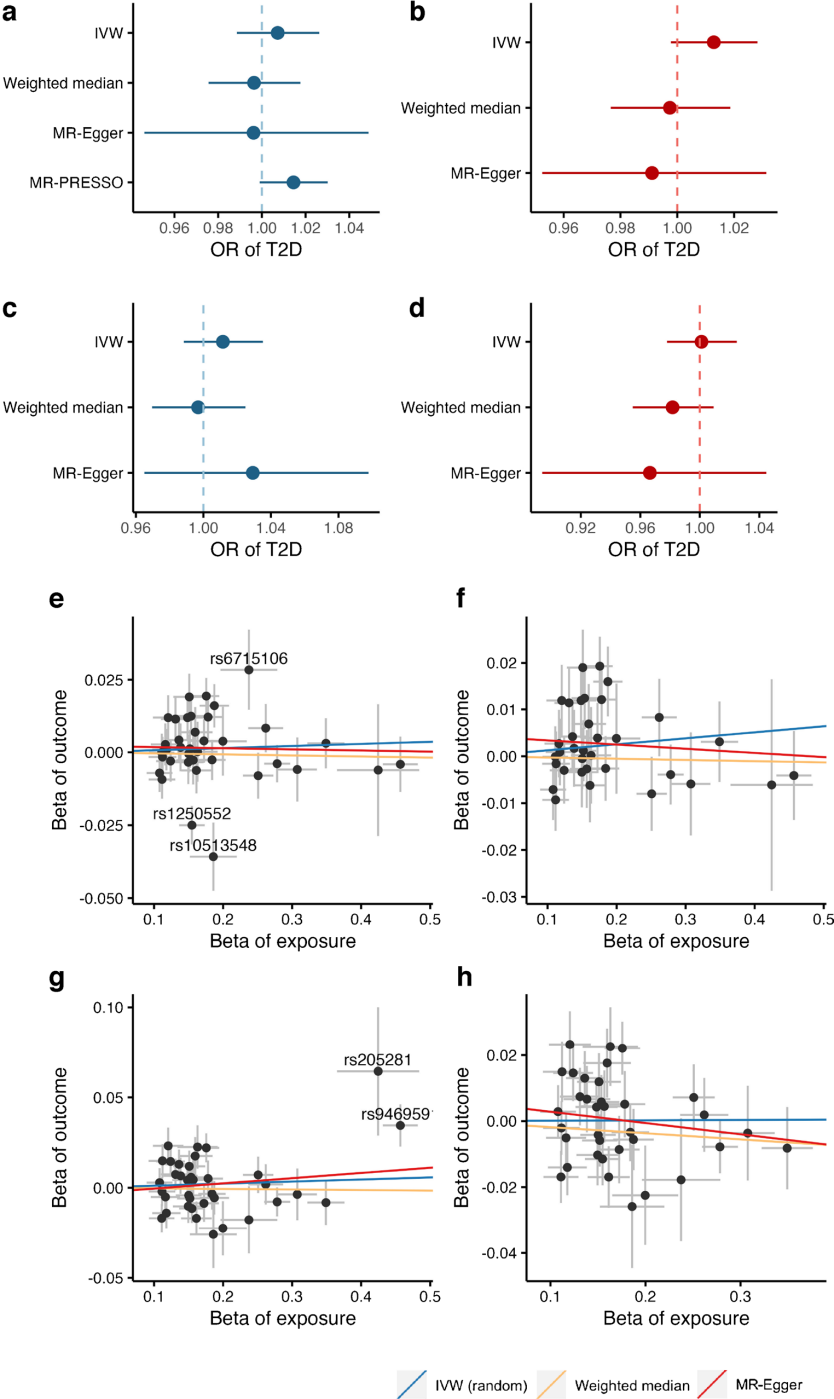
Association between genetic liability to coeliac disease and risk of type 2 diabetes (T2D). (**a**,**b**) Forest plots of MR association based on all SNPs in DIAGRAM (**a**) and after removing three outlying SNPs in DIAGRAM (**b**). (**c**,**d**) Forest plot of MR association based on all SNPs in FinnGen (**c**) and after removing two outlying SNPs in FinnGen (**d**). (**e**,**f**) Scatter plot of MR association based on all SNPs in DIAGRAM (**e**) and after removing three outlying SNPs in DIAGRAM (**f**). (**g**,**h**) Scatter plot of MR association based on all SNPs in FinnGen (**g**) and after removing two outlying SNPs in FinnGen (**h**). Outlier SNPs were identified by the scatter plots (labelled in **e** and **g**). In the analysis including all SNPs in DIAGRAM, we showed estimates for MR-PRESSO after the removal of two SNP outliers (rs10513548 and rs1250552), which were also identified by the scatter plot. The MR-PRESSO result was not available for other analyses due to no outlier being detected. In this case, the estimate of MR-PRESSO is identical to that of inverse variance weighted (IVW) random effects. Key applies to (**e**–**h**)

Genetic liability to coeliac disease was not associated with the risk of type 2 diabetes in either the DIAGRAM consortium (OR 1.01 [95% CI 0.99, 1.03]; *p*=0.451) or the FinnGen study (OR 1.01 [95% CI 0.99, 1.04]; *p*=0.331) (Fig. 3a–d and ESM Table 7). Even though we observed moderate heterogeneity for the associations, there was no indication of directional pleiotropy by MR-Egger intercept test (*p*>0.05) and the association remained null in the sensitivity analyses (ESM Table 7). The scatter plot identified three and two SNP outliers in the analysis in DIAGRAM and FinnGen, respectively (Fig. 3e,g). The null association remained in the analysis after removal of these outlying SNPs (Fig. 3f,h). We did not identify any association between genetic liability to coeliac disease and any studied glycaemic traits (ESM Table 8).

## Discussion

This nationwide matched cohort including 46,150 individuals with coeliac disease and 219,763 comparator individuals found no association between coeliac disease and the risk of incident type 2 diabetes. This null finding was also observed in the case–control analysis that examined the risk of type 2 diabetes prior to coeliac disease diagnosis. Among individuals with coeliac disease who had a second biopsy, persistent villous atrophy was not associated with an increased risk of type 2 diabetes compared with mucosal healing. Our results confirm the findings of an earlier neutral study [12] but add to that paper through absolute and relative risk estimates, the use of matched comparator individuals, and larger study samples that enabled subgroup analyses. Finally, our null results were confirmed in an MR analysis with a total of 122,747 type 2 diabetes cases where genetic liability to coeliac disease was not associated with type 2 diabetes.

### Comparison with previous studies

Type 2 diabetes is a metabolic disorder and makes up >90% of all diabetes mellitus cases [34]. It tends to occur in middle and old age [34] and is linked to poor diet [35], low levels of physical exercise [36, 37] and overweight [38]. Characteristics of individuals with type 2 diabetes argue against a positive relationship with coeliac disease, where individuals typically have a younger age at onset [5] and are more often underweight [39, 40], but other factors such a possible presence of inflammation in both coeliac disease and type 2 diabetes (important for the pathogenesis of type 2 diabetes [38]) may argue for a positive association.

Overall, we found no association with type 2 diabetes, consistent with the study by Kylökäs et al [12]. That study examined patient charts (sometimes supplemented with interviews) of 1398 adult individuals with coeliac disease [12]. Patients were then compared with individuals from a separate health survey (diabetes in that study was self-reported but confirmed in primary care). Kylökäs et al reported a type 2 diabetes prevalence in coeliac disease of 2.8% compared with 3.0% in the reference population [12]. In our study, even though the overall cumulative incidence rate of type 2 diabetes was only around 0.8% in coeliac disease and in the comparator group after 15 years of follow up, our nationwide cohort involved a comparatively large proportion of children with coeliac disease when compared with the study by Kylökäs et al [12]. For those diagnosed with coeliac disease and those in the comparator group aged ≥60 years, the cumulative incidence rate for both was 1.9%. In contrast to both our study and that of Kylökäs et al [12], Kabbani et al [13] reported a lower type 2 diabetes prevalence in participants with coeliac disease than in control participants (3.5% vs 9.6%% in internal control participants and 9.8% in an external control population retrieved from the NHANES cohort). Adjusting for confounders, the corresponding OR in their study was 0.49 (95% CI 0.29, 0.83) [13]. Most of the type 2 diabetes cases were diagnosed prior to coeliac disease; however, no OR was reported specifically for pre-existing type 2 diabetes. Using Swedish ESPRESSO data, we found a robust null association also between coeliac disease and pre-existing type 2 diabetes (OR 1.04 [95% CI 0.98, 1.10]). We further examined the causality of any association by use of MR analysis based on two large-scale studies, and this confirmed a lack of association with type 2 diabetes.

We speculate that one of the reasons for the inverse relationship in the study by Kabbani et al [13] is concerned with patient selection. If that study had a high proportion of participants with malabsorption and low weight (due to lower coeliac disease awareness in a US setting followed by a probable low number of diagnosed cases among average weight/overweight individuals), it may therefore have had a higher proportion of highly symptomatic individuals (underweight) presenting for healthcare. This may explain the substantially lower risk of type 2 diabetes in a US population. A paper with data on the clinical presentation of individuals with coeliac disease in Olmsted County, USA, indicated that weight loss went from being a common feature at diagnosis in around 20% of patients in the year 2000 down to <5% in 2010 [41]. This is supported by the fact that weight loss at coeliac disease diagnosis in the study by Kabbani et al was more prevalent in individuals with coeliac disease without type 2 diabetes, and less common in future type 2 diabetes patients [13]. In contrast, a lower proportion of Swedish patients may have suffered from malabsorption and classical coeliac disease [42]. Unfortunately, we did not have data on symptoms or BMI and were unable to examine the impact of any interaction between BMI and coeliac disease on the risk of type 2 diabetes.

Consistent with Kabbani et al (the *p* value for enteropathy on second biopsy and type 2 diabetes was 0.5) [13], we found no association between persistent villous atrophy and type 2 diabetes. Persistent villous atrophy is strongly linked to ongoing inflammation which has otherwise been implicated in the pathogenesis of type 2 diabetes [38, 43]. However, we did not find an increased risk of type 2 diabetes among those with persistent villous atrophy, which indicates that in coeliac disease, underlying inflammation is unlikely to drive type 2 diabetes development.

### Strengths and clinical implications

Among the strengths of our study was the high validity of our coeliac disease definition and our use of nationwide register data to identify cases of type 2 diabetes. We used both ICD codes and ATC records to increase sensitivity for type 2 diabetes diagnosis. We removed any potentially ambiguous diagnosis of type 2 diabetes to increase the specificity. During a follow-up of more than 15 years, some 6000 individuals with coeliac disease developed type 2 diabetes (as a comparison, five individuals with a coeliac disease diagnosis who had commenced a gluten-free diet had a subsequent type 2 diabetes diagnosis in the Kabbani et al study) [13]. Our large numbers yielded an unprecedented statistical power and allowed us to study men vs women (no difference), calendar year patterns (none), and risk according to follow-up duration (no difference). While there was a small reduction in risk for type 2 diabetes among individuals with coeliac disease diagnosed at age 40–<60 years, no association among those diagnosed at ≥60 years was observed. This contrasts with the Finnish study, where a lower type 2 diabetes prevalence was found in men aged ≥65 years [12]. We urge caution when interpreting these data due to inconsistent results and borderline statistical significance. Our null results were robust in a sensitivity analysis when we excluded individuals with diabetes insipidus and nephrogenic diabetes, gestational diabetes or Cushing’s syndrome.

Our findings have some clinical implications. First, our data suggest that it is not necessary to single out individuals with coeliac disease for type 2 diabetes screening. Second, the lack of association with type 2 diabetes argues against the metabolic disorder playing a major role in the excess risk of CVD in coeliac disease noted in some studies [44], or the potentially poorer prognosis after myocardial infarction in coeliac disease [45]. However, the findings cannot exclude the possibility that type 2 diabetes may interact with coeliac disease on the risk of CVD. In addition, we calculated the absolute risk of type 2 diabetes, where for instance the incidence rate among people diagnosed with coeliac disease aged 40–<60 and ≥60 years was 12/1000 and 21/1000 person-years (equal to 1 in 8 and 1 in 5 patients during a 10 year period), respectively. These data can help physicians inform individuals with coeliac disease about the risk of future type 2 diabetes.

### Limitations

Our study has some limitations. We cannot fully rule out that a minimal inverse relationship was hidden by a relative increase in type 2 diabetes detected through surveillance bias of participants with coeliac disease in our study. Without data on BMI, physical activity, smoking and diet, we cannot minimise corresponding confounding effects. We examined type 2 diabetes according to persistent villous atrophy vs mucosal healing in individuals with coeliac disease but did not study actual gluten consumption or dietary adherence. Finally, we cannot rule out that our findings have limited generalisability since Sweden has a relatively homogenous ethnic population and our MR analysis was confined to European ancestry.

In summary, nationwide population-based observational and two-sample MR analyses found no association between coeliac disease and type 2 diabetes.

### Supplementary Information

Below is the link to the electronic supplementary material.

ESM 1 (PDF 385 KB)

## Data Availability

Due to Swedish regulations, the data in this study cannot be shared by the authors. However, the data can be requested from the two government agencies: the National Board of Health and Welfare (socialstyrelsen@socialstyrelsen.se), and Statistics Sweden (scb@scb.se). Histopathology data can be obtained through Swedish pathology departments. On request, JFL can provide a list of the departments and their contact details. Data used for MR are publicly available and corresponding papers have been cited.

## Abbreviations

ATC: Anatomical Therapeutic Chemical
CCI: Charlson Comorbidity Index
DIAGRAM: DIAbetes Genetics Replication And Meta-analysis
ESPRESSO: Epidemiology Strengthened by histoPathology Reports in Sweden
LISA: Longitudinal integrated database for health insurance and labour market studies
MAGIC: Meta-Analyses of Glucose and Insulin-related traits Consortium
MR: Mendelian randomisation
NPR: (Swedish) National Patient Register
PDR: (Swedish) Prescribed Drug Register
