# Supplementary material for: Coeliac disease and type 2 diabetes risk: a nationwide matched cohort and Mendelian randomisation study
Source: Diabetologia. 2024 May 21;67(8):1630–41. doi: 10.1007/s00125-024-06175-8 (PMC11343898; doi:10.1007/s00125-024-06175-8)
Supplement: Supplementary file 1 — ESM 1 (PDF 385 KB) [file 125_2024_6175_MOESM1_ESM.pdf]

Electronic supplementary material

Celiac Disease and Type 2 Diabetes Risk: A Nationwide Matched Cohort and Mendelian Randomization Study

Shuai Yuan, Dan Leffler, Benjamin Lebwohl, Peter HR Green, Jiangwei Sun, Sofia Carlsson, Susanna C. Larsson, Jonas F Ludvigsson

Table of Contents

ESM Table 1. Topography and SnoMed characteristics for celiac disease diagnosis................. 2

ESM Table 2. Diagnostic codes for type 2 diabetes and other disease definitions..................... 3

ESM Table 3. Genetic instruments for celiac disease and associated with type 2 diabetes in DIAGRAM and FinnGen consortia. 4

ESM Table 4. Diagnosis of type 2 diabetes in the DIAGRAM consortium and FinnGen study..... 6

ESM Table 5. Baseline characteristics of persistent villous atrophy and mucosal healing among patients with celiac disease. 8

ESM Table 6. Association between persistent villous atrophy and the hazard ratio of incident type 2 diabetes. 9

ESM Table 7. Genetic liability to celiac disease in relation to the risk of type 2 diabetes. 10

ESM Table 8. Genetic liability to CeD in relation to four glycemic traits................................ 11

ESM Fig. 1. Cumulative incidence of type 2 diabetes for patients with celiac disease (CeD) versus matched comparators with numbers at risk shown below the graph. 12

ESM Table 1. Topography and SnoMed characteristics for celiac disease diagnosis

Topography	SnoMed code
all T64, only T65, T65000 and T651 (not T652-9 (ileum))	D6218, D62180, D62188, D6218X, D6218Y, M58, M5800, M58000, M58001, M58005, M58006, M58007

ESM Table 2. Diagnostic codes for type 2 diabetes and other disease definitions

Diagnosis	ICD-7 (1964-1968)	ICD-8 (1969-1986)	ICD-9 (1987-1996)	ICD-10 (1997-)	ATC
Type 2 diabetes	260	250	250	E11	A10B
Type 1 diabetes	260 (≤30 years*)	250 (≤30 years*)	250 (≤30 years*)	E10 (no age restriction)	A10A before age ≤30 years* or prescribed within one month since first E10-E14
Other diabetes	-	-	-	E12-14	
Pancreatitis (acute or chronic)	587	577	577	K85; K86	
Pancreatic insufficiency					A09AA02
Pancreatic cancer	157	157	157	C25	
Diabetes insipidus and nephrogenic diabetes	272,40; 260,30	253; 273,81	253F; 588B	E23.2; N25.1	
Gestational diabetes				O24	
Cushing's syndrome	277,10	258	255A	E24	

ICD, International Classification of Disease; ATC, Anatomical Therapeutic Chemical.
*Refers to age of first diagnosis

ESM Table 3. Genetic instruments for celiac disease and associated with type 2 diabetes in DIAGRAM and FinnGen consortia.

					Celiac disease			Type 2 diabetes		
Source	SNP	EA	NEA	EAF	Beta	SE	Pval	Beta	SE	Pval
DIAGRAM	rs1018326	C	T	0.56	0.152	0.019	3.06E-16	-0.006	0.008	0.458
DIAGRAM	rs1050976	T	C	0.52	-0.111	0.018	1.84E-09	0.017	0.008	0.032
DIAGRAM	rs10513548	C	A	0.90	-0.186	0.034	4.23E-08	0.026	0.019	0.166
DIAGRAM	rs10800746	T	C	0.71	-0.112	0.020	2.57E-08	-0.015	0.009	0.104
DIAGRAM	rs10892258	A	G	0.23	-0.150	0.022	1.73E-11	0.010	0.009	0.264
DIAGRAM	rs1107943	C	T	0.94	0.200	0.035	7.95E-09	-0.022	0.015	0.135
DIAGRAM	rs11801183	T	C	0.82	-0.138	0.025	1.69E-08	-0.007	0.010	0.508
DIAGRAM	rs11851414	C	T	0.21	0.120	0.022	4.71E-08	0.023	0.010	0.021
DIAGRAM	rs11875687	C	T	0.14	0.160	0.025	1.92E-10	0.018	0.010	0.093
DIAGRAM	rs12068671	C	T	0.20	-0.157	0.024	1.40E-10	-0.004	0.011	0.684
DIAGRAM	rs1250552	G	A	0.44	-0.155	0.019	7.97E-17	0.011	0.008	0.149
DIAGRAM	rs13003464	G	A	0.64	0.154	0.019	4.34E-16	0.006	0.008	0.479
DIAGRAM	rs13132308	G	A	0.84	-0.349	0.027	1.87E-38	0.008	0.012	0.510
DIAGRAM	rs1323292	A	G	0.18	0.262	0.025	4.23E-25	0.002	0.011	0.867
DIAGRAM	rs1378938	C	T	0.32	-0.118	0.020	7.79E-09	0.014	0.008	0.096
DIAGRAM	rs17264332	G	A	0.83	0.251	0.022	4.98E-30	0.007	0.010	0.477
DIAGRAM	rs182429	G	A	0.56	-0.150	0.019	8.49E-16	0.004	0.008	0.599
DIAGRAM	rs1893592	C	A	0.72	-0.124	0.021	2.96E-09	-0.015	0.009	0.087
DIAGRAM	rs1980422	T	C	0.26	-0.172	0.022	1.43E-15	0.009	0.010	0.398
DIAGRAM	rs2030519	A	G	0.47	0.278	0.019	3.00E-49	-0.008	0.008	0.330
DIAGRAM	rs205281	C	T	0.98	0.425	0.060	9.84E-13	0.064	0.036	0.070
DIAGRAM	rs2097282	T	C	0.31	-0.184	0.020	1.13E-20	0.003	0.009	0.698
DIAGRAM	rs3184504	C	T	0.54	-0.176	0.019	5.42E-21	-0.022	0.008	0.006
DIAGRAM	rs4445406	C	T	0.33	-0.136	0.020	5.42E-12	-0.013	0.008	0.113
DIAGRAM	rs4821124	C	T	0.18	0.151	0.023	5.72E-11	0.012	0.009	0.172
DIAGRAM	rs55743914	T	C	0.77	0.187	0.021	1.14E-18	-0.006	0.009	0.547
DIAGRAM	rs61579022	A	G	0.39	0.108	0.019	9.92E-09	0.003	0.008	0.722
DIAGRAM	rs61907765	T	C	0.24	0.161	0.022	3.43E-13	-0.017	0.009	0.069
DIAGRAM	rs6498114	T	G	0.24	-0.131	0.021	5.83E-10	-0.007	0.009	0.396
DIAGRAM	rs6715106	G	A	0.95	-0.237	0.041	8.38E-09	0.018	0.019	0.340
DIAGRAM	rs7104791	C	T	0.79	-0.148	0.022	1.89E-11	-0.004	0.009	0.628
DIAGRAM	rs744254	A	G	0.73	0.116	0.021	3.04E-08	-0.005	0.008	0.539
DIAGRAM	rs7616215	T	C	0.36	-0.112	0.019	7.27E-09	0.002	0.008	0.799
DIAGRAM	rs76830965	A	C	0.08	0.307	0.028	2.57E-27	-0.004	0.014	0.799
DIAGRAM	rs79758729	G	A	0.89	0.163	0.029	2.12E-08	0.023	0.012	0.059
DIAGRAM	rs9469591	C	T	0.12	0.457	0.025	1.10E-72	0.034	0.012	0.003
DIAGRAM	rs990171	C	A	0.78	-0.178	0.022	1.22E-16	-0.005	0.010	0.611
FinnGen	rs1018326	C	T	0.56	0.152	0.019	3.06E-16	0.001	0.007	0.853
FinnGen	rs1050976	T	C	0.52	-0.111	0.018	1.84E-09	0.000	0.007	0.988
FinnGen	rs10513548	C	A	0.90	-0.186	0.034	4.23E-08	0.036	0.012	0.002
FinnGen	rs10800746	T	C	0.71	-0.112	0.020	2.57E-08	0.002	0.007	0.817
FinnGen	rs10892258	A	G	0.23	-0.150	0.022	1.73E-11	0.003	0.008	0.652
FinnGen	rs1107943	C	T	0.94	0.200	0.035	7.95E-09	0.004	0.012	0.748
FinnGen	rs11801183	T	C	0.82	-0.138	0.025	1.69E-08	-0.002	0.008	0.835
FinnGen	rs11851414	C	T	0.21	0.120	0.022	4.71E-08	0.012	0.008	0.125
FinnGen	rs11875687	C	T	0.14	0.160	0.025	1.92E-10	0.007	0.009	0.422
FinnGen	rs12068671	C	T	0.20	-0.157	0.024	1.40E-10	0.003	0.008	0.744

FinnGen	rs1250552	G	A	0.44	-0.155	0.019	7.97E-17	0.025	0.007	0.000
FinnGen	rs13003464	G	A	0.64	0.154	0.019	4.34E-16	0.012	0.007	0.060
FinnGen	rs13132308	G	A	0.84	-0.349	0.027	1.87E-38	-0.003	0.009	0.718
FinnGen	rs1323292	A	G	0.18	0.262	0.025	4.23E-25	0.008	0.008	0.316
FinnGen	rs1378938	C	T	0.32	-0.118	0.020	7.79E-09	-0.001	0.007	0.899
FinnGen	rs17264332	G	A	0.83	0.251	0.022	4.98E-30	-0.008	0.008	0.308
FinnGen	rs182429	G	A	0.56	-0.150	0.019	8.49E-16	0.001	0.007	0.939
FinnGen	rs1893592	C	A	0.72	-0.124	0.021	2.96E-09	0.003	0.007	0.674
FinnGen	rs1980422	T	C	0.26	-0.172	0.022	1.43E-15	-0.004	0.008	0.605
FinnGen	rs2030519	A	G	0.47	0.278	0.019	3.00E-49	-0.004	0.006	0.541
FinnGen	rs205281	C	T	0.98	0.425	0.060	9.84E-13	-0.006	0.023	0.787
FinnGen	rs2097282	T	C	0.31	-0.184	0.020	1.13E-20	0.003	0.007	0.707
FinnGen	rs3184504	C	T	0.54	-0.176	0.019	5.42E-21	-0.019	0.006	0.002
FinnGen	rs4445406	C	T	0.33	-0.136	0.020	5.42E-12	-0.004	0.007	0.543
FinnGen	rs4821124	C	T	0.18	0.151	0.023	5.72E-11	0.019	0.008	0.018
FinnGen	rs55743914	T	C	0.77	0.187	0.021	1.14E-18	0.016	0.008	0.034
FinnGen	rs61579022	A	G	0.39	0.108	0.019	9.92E-09	-0.007	0.007	0.274
FinnGen	rs61907765	T	C	0.24	0.161	0.022	3.43E-13	-0.006	0.008	0.430
FinnGen	rs6498114	T	G	0.24	-0.131	0.021	5.83E-10	-0.011	0.008	0.131
FinnGen	rs6715106	G	A	0.95	-0.237	0.041	8.38E-09	-0.028	0.014	0.040
FinnGen	rs7104791	C	T	0.79	-0.148	0.022	1.89E-11	-0.012	0.008	0.140
FinnGen	rs744254	A	G	0.73	0.116	0.021	3.04E-08	0.003	0.007	0.709
FinnGen	rs7616215	T	C	0.36	-0.112	0.019	7.27E-09	0.009	0.007	0.159
FinnGen	rs76830965	A	C	0.08	0.307	0.028	2.57E-27	-0.006	0.011	0.592
FinnGen	rs79758729	G	A	0.89	0.163	0.029	2.12E-08	0.000	0.010	0.984
FinnGen	rs9469591	C	T	0.12	0.457	0.025	1.10E-72	-0.004	0.010	0.667
FinnGen	rs990171	C	A	0.78	-0.178	0.022	1.22E-16	-0.012	0.008	0.113

EA, effect allele; EAF, effect allele frequency; NEA, non-effect allele; SE, standard error; SNPs, single nucleotide polymorphisms.

ESM Table 4. Diagnosis of type 2 diabetes in the DIAGRAM consortium and FinnGen study.

Study name	Case ascertainment
BioMe Biobank	Random glucose ≥200mg/dl ever, physician-entered diagnosis (at least two occurrences on two separate days) ever, or T2D medication (at least two occurrences on two separate days) ever.
deCODE Genetics	Diagnostic fasting glucose or HbA1c levels, hospital discharge diagnosis, use of oral diabetes medication or self-report.
Diabetes Gene Discovery Group	Hospital diagnosis based on HbA1c and fasting glucose.
Diabetes Genetics Initiative	WHO (1999) criteria with fasting plasma glucose ≥7.0mmol/l or a 2-hour glucose ≥11.1mmol/l during an oral glucose tolerance test; age at onset >35 years and no detectable GAD Ab; no family history of MODY mutation carriers.
Estonian Genome Center of the University of Tartu	Previous T2D diagnosis.
European Prospective Investigation into Cancer and Nutrition	Incident T2D based on: self-report (self-reported T2D, doctor diagnosed T2D, diabetes drug use); linkage to primary care registers; secondary care registers, medication use (drug registers); hospital admissions; mortality data; local and national diabetes and pharmaceutical registers (Denmark and Sweden).
Framingham Heart Study	On T2D treatment or fasting glucose ≥7mmol/l (when available) or 2-hour glucose ≥11.1mmol/l.
Finland-United States Investigation of NIDDM Genetics	WHO 1999 criteria of fasting glucose ≥7.0mmol/l or 2-hour plasma glucose ≥11.1mmol/l or reported diabetes medication use or based on medical record review; no known or probable T1D among first degree relatives; excluded if insulin treatment initiated within 10 years of disease diagnosis, detectable levels of anti-GAD antibodies and fasting C-peptide ≤0.30nmol/l; excluded if insulin treatment initiated within 4 years of diagnosis and fasting C-peptide ≤0.30nmol/l.
German Chronic Kidney Disease	Anti-diabetic medication (ATC code A10*) or HbA1c ≥6.5%.
Genetic Epidemiology Network of Arteriosclerosis	Use of T2D medications or fasting glucose ≥7.0mmol/l.
Resource for Genetic Epidemiology on Adult Health and Aging	ICD9 codes.
Genetics of Diabetes and Audit Research in Tayside Scotland	Electronic medical records.
Genetic Overlap between Metabolic and Psychiatric traits & TEENs of Attica: Genes and Environment	Previous diagnosis of T2D with or without psychiatric disease.
Health Professionals' Follow-Up Study	Self-reported diabetes confirmed by a validated supplementary questionnaire (National Diabetes Data Group criteria before 1998; American Diabetes Association diagnostic criteria from 1998 onwards).
Collaborative Health Research in the Region of Augsburg	Self-reported.
Multi-Ethnic Study of Atherosclerosis	Known T2D or fasting whole-blood glucose ≥7mmol/l.
Metabolic Syndrome in Men	WHO 1999 criteria of fasting glucose ≥7.0mmol/l or 2-hour plasma glucose ≥11.1mmol/l or reported diabetes medication use or based on medical record review; no known or probable T1D among first degree relatives; excluded if insulin treatment initiated within 10 years of disease diagnosis, detectable levels of anti-GAD

	antibodies and fasting C-peptide ≤0.30nmol/l; excluded if insulin treatment initiated within 4 years of diagnosis and fasting C-peptide ≤0.30nmol/l.
Mass General Brigham Biobank	Curated disease algorithm PPV >99% and age of at least 30.
Michigan Genomics Initiative	EHR-derived ICD-9 codes: 250.00, 250.02, 250.20, 250.22, 250.30, 250.32, 250.80, 250.82, 250.90, 250.92.
Netherlands Epidemiology of Obesity	Fasting glucose ≥7.0mmol/l, or physician-diagnosed diabetes, or on diabetes treatment.
Nurses' Health Study	Self-reported diabetes confirmed by a validated supplementary questionnaire (National Diabetes Data Group criteria before 1998; American Diabetes Association diagnostic criteria from 1998 onwards).
Northwestern University Genetics	ICD9 codes (excluding those with ketoacidosis codes), excluding individuals treated only with insulin and have never been on a T2D medication; or individuals with HbA1c ≥6.5, fasting glucose >125mg/dl, or random glucose >200mg/dl, and prescribed T2D medication.
Prospective Investigation of the Vasculature in Uppsala Seniors	Known T2D or fasting whole blood glucose >6.1mmol/l.
PROspective Study of Pravastatin in the Elderly at Risk	Known diabetes mellitus or fasting blood glucose >7mmol/l.
Rotterdam Study	WHO guidelines: fasting blood glucose >7.0 mmol/l or use of blood-glucose-lowering medication (derived from both structured home interviews and linkage to pharmacy records).
Danish T2D case-control study	Self-report, anti-diabetic treatment, fasting plasma glucose >7.0mmol/l or 2-hr plasma glucose >11.1mmol/l.
UK Biobank	Self-reported medical history and relevant medication.
Uppsala Longitudinal Study of Adult Men	Hospital discharge register-defined diabetes before 2002.
Wellcome Trust Case Control Consortium	Prescribed treatment with sulphonylureas, biguanides, other oral agents and/or insulin, or in the case of individuals treated with diet alone, historical or contemporary laboratory evidence of hyperglycemia.
The FinnGen study	ICD-9 250.A; ICD-10 E11.

ICD, International Classification of Disease.

ESM Table 5. Baseline characteristics of persistent villous atrophy and mucosal healing among patients with celiac disease.

Characteristic	Category	Persistent villous atrophy (N = 2685)	Mucosal healing (N = 6368)
Sex	Women	1604 (59.7%)	4171 (65.5%)
	Men	1081 (40.3%)	2197 (34.5%)
Age, years	Mean ± SD	40.7 ± 24.2	29.6 ± 22.2
	Median (IQR)	44.0 (21.0 to 61.0)	28.0 (9.0 to 47.0)
Age group	<18 years	584 (21.8%)	2218 (34.8%)
	≥18-<40 years	573 (21.3%)	1987 (31.2%)
	≥40-<60 years	810 (30.2%)	1414 (22.2%)
	≥60 years	718 (26.7%)	749 (11.8%)
Country of birth	Sweden	2476 (92.2%)	5944 (93.3%)
	Other Nordic countries	87 (3.2%)	138 (2.2%)
	Rest of World	122 (4.5%)	286 (4.5%)
Education levels*	≤ 9 years	551 (20.5%)	624 (9.8%)
	10-≤12 years	882 (32.8%)	1732 (27.2%)
	≥13 years	1234 (46%)	3987 (62.6%)
	Missing	18 (0.7%)	25 (0.4%)
Start year of follow-up	1978-1999	1106 (41.2%)	1990 (31.2%)
	2000-2017	1579 (58.8%)	4378 (68.8%)
Charlson Comorbidity Index	0	2182 (81.3%)	5391 (84.7%)
	1	240 (8.9%)	506 (7.9%)
	2	125 (4.7%)	180 (2.8%)
	≥3	138 (5.1%)	291 (4.6%)

IQR, interquartile range. * The highest documented education level was referenced for each participant, while for children (<18 years old), we considered the highest educational attainment of their parents.

ESM Table 6. Association between persistent villous atrophy and the hazard ratio of incident type 2 diabetes.

Participants	Persistent villous atrophy		Mucosal healing		HR	95% CI
	T2D Cases	Person-years	T2D Cases	Person-years		
Overall	398	41419	745	96629	1.02	0.9, 1.16
Sex						
Men	164	15642	257	33495	0.89	0.72, 1.1
Women	234	25777	488	63134	1.09	0.92, 1.28
Diagnosis age						
< 18 years	53	12647	206	41767	1.02	0.74, 1.4
≥18-<40 years	46	9274	123	27586	0.92	0.66, 1.28
≥40-<60 years	164	12506	260	19911	1.01	0.83, 1.23
≥60 years	135	6992	156	7365	1.17	0.91, 1.5
Start year of follow-up						
1969-1999	204	24729	310	46964	1.06	0.87, 1.3
2000-2017	194	16690	435	49665	1.00	0.84, 1.18
Follow-up duration						
< 1 year	17	29	38	37	1.60	0.75, 3.42
1-5 years	83	616	166	995	1.06	0.78, 1.44
5-10 years	99	4490	222	11555	1.13	0.87, 1.48
10-20 years	149	15429	258	41205	0.96	0.78, 1.19
>20 years	50	20855	61	42837	0.81	0.54, 1.2

CI, confidence interval; HR, hazard ratio. The analysis was adjusted for age, sex, calendar year, and county of residence, education levels, country of birth, and the Charlson Comorbidity Index.

ESM Table 7. Genetic liability to celiac disease in relation to the risk of type 2 diabetes.

Source	Cases	Controls	SNPs	Method	OR / Beta*	95% CI	*P*
DIAGRAM	80,154	853,816	37	IVW-random effects	1.01	0.99, 1.03	0.451
				Weighted median	1.00	0.98, 1.02	0.737
				MR-Egger	1.00	0.95, 1.05	0.885
				MR-Egger (intercept)	0.00	-0.01, 0.01	0.654
				MR-PRESSO	1.01	1.00, 1.03	0.075
FinnGen	42,593	337,038	37	IVW-random effects	1.01	0.99, 1.04	0.331
				Weighted median	1.00	0.97, 1.02	0.826
				MR-Egger	1.03	0.97, 1.1	0.386
				MR-Egger (intercept)	0.00	-0.01, 0.01	0.575
				MR-PRESSO	-	-	-

Beta shown for MR-Egger (intercept). CI, confidence interval; DIAGRAM, DIAbetes Genetics Replication And Meta-analysis; IVW, inverse variance weighted; OR, odds ratio; IVs, instrumental variables; SNPs, single nucleotide polymorphisms. We showed estimates for MR-PRESSO after the removal of SNP outliers. The MR-PRESSO result was not available for FinnGen due to no outlier detected. In this case, the estimate of MR-PRESSO is identical to that of IVW-random effects. The Cochran's Q = 68 for DIAGRAM and 65 for FinnGen.

ESM Table 8. Genetic liability to CeD in relation to four glycemic traits.

Outcome	SNPs	Cochran's Q	$P_{MR\text{-}Egger\ intercept}$	Inverse variance weighted			Weighted median		
				**Beta**	**95% CI**	***P***	**Beta**	**95% CI**	***P***
2hGlu	37	30	0.876	-0.008	-0.025, 0.009	0.349	-0.008	-0.035, 0.019	0.557
FG	37	62	0.842	0.001	-0.004, 0.006	0.570	-0.001	-0.007, 0.006	0.808
FI	37	75	0.514	0.002	-0.004, 0.009	0.454	0.001	-0.005, 0.008	0.666
HbA1c	37	89	0.079	-0.001	-0.006, 0.003	0.514	0.002	-0.002, 0.007	0.338
				**MR-Egger**			**MR-PRESSO**		
				**Beta**	**95% CI**	***P***	**Beta**	**95% CI**	***P***
2hGlu	37	30	0.876	-0.004	-0.054, 0.045	0.867	-	-	-
FG	37	62	0.842	0.003	-0.011, 0.017	0.697	0.000	-0.004, 0.004	0.923
FI	37	75	0.514	-0.003	-0.021, 0.014	0.731	0.001	-0.004, 0.006	0.628
HbA1c	37	89	0.079	0.009	-0.003, 0.02	0.160	0.000	-0.003, 0.004	0.829

2hGlu, 2-h glucose after an oral glucose challenge; HbA1c, glycated hemoglobin; CI, confidence interval; FG, fasting glucose; FI, fasting insulin; SNPs, single nucleotide polymorphisms.

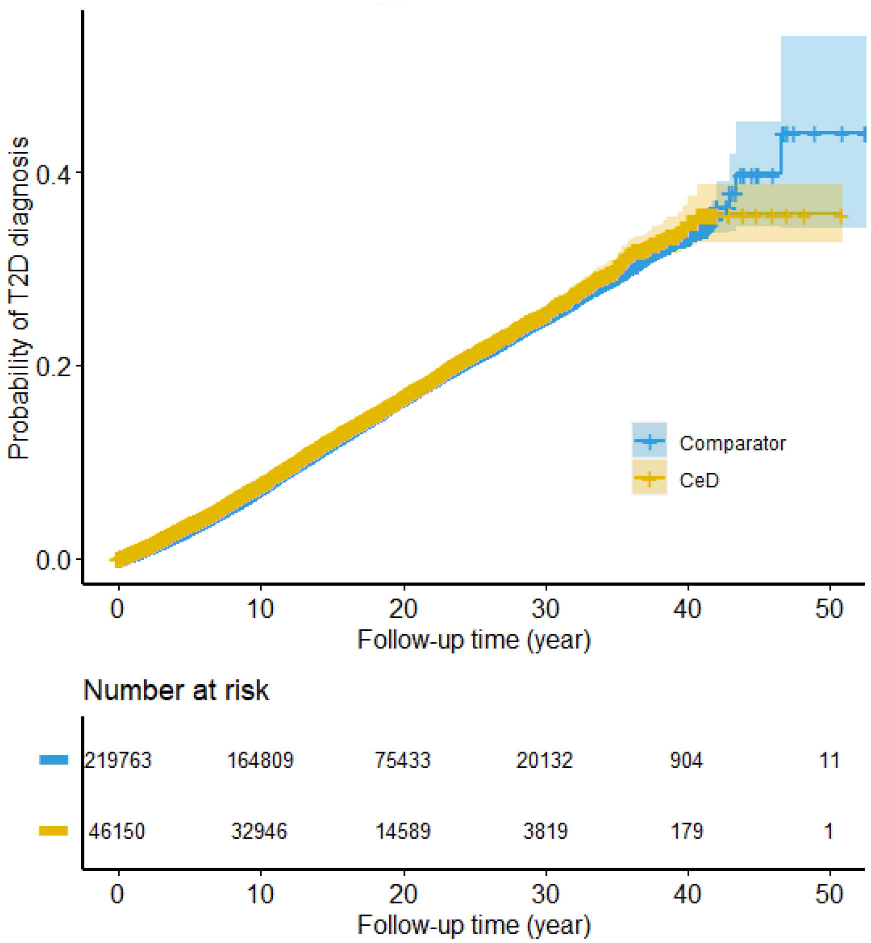

ESM Fig. 1. Cumulative incidence of type 2 diabetes for patients with celiac disease (CeD) versus matched comparators with numbers at risk shown below the graph.
